# Small RNA trafficking at the forefront of plant–pathogen interactions

**DOI:** 10.12688/f1000research.15761.1

**Published:** 2018-10-11

**Authors:** Yan Zhao, Xiangxiu Liang, Jian-Min Zhou

**Affiliations:** 1 State Key Laboratory of Plant Genomics, Institute of Genetics and Developmental Biology, Chinese Academy of Sciences, Beijing, China

**Keywords:** Extracellular vesicles, Plant immunity, Small RNA, Cross-kingdom trafficking

## Abstract

Plants and pathogenic microbes are engaged in constant attacks and counterattacks at the interface of the interacting organisms. Much of the molecular warfare involves cross-kingdom trafficking of proteins, nucleic acids, lipids, and metabolites that act as toxins, inhibitors, lytic enzymes, and signaling molecules. How various molecules are transported across the boundaries of plants and pathogens has remained largely unknown until now. Extracellular vesicles have emerged as likely carriers of molecular ammunition for both plants and pathogens. Recent advances are beginning to show how extracellular vesicles serve as powerful vehicles that transfer small RNAs from plants to fungal cells to diminish pathogen virulence and from fungi to plant cells to dampen host immunity.

## Introduction

The site of plant–pathogen contact is the frontline where the two battling organisms exchange numerous molecules as ammunition. Plant cells can secrete lytic enzymes, antimicrobial proteins, peptides, and metabolites to fend off pathogens. Likewise, pathogens can secrete a repertoire of effector proteins and metabolites that suppress plant immunity or manipulate plant physiology to promote pathogenesis ^1, 2^.

Work in the last decade has uncovered active exchanges of small RNAs (sRNA) between host plants and pathogenic microbes ^3– 7^. sRNAs are known to play major roles in plant resistance and microbial pathogenesis ^8^. Both pathogens and host plants encode sRNAs that are targeted to genes of their counterparts for silencing, a phenomenon referred to as cross-kingdom RNA interference (RNAi) ^9, 10^.

Cross-kingdom trafficking is a vibrant research area in plant–microbe interactions. Bacteria possess multiple classes of secretion systems. For example, through the type I secretion system, bacteria secrete molecules of a diverse nature, from ions and metabolites to proteins of various sizes. Gram-negative bacterial pathogens use the type III secretion system to deliver specific effector proteins directly into host cells. The type IV system can deliver proteins and nucleic acids into host cells. As a major route of secretion in eukaryotes, the conventional secretory pathway secretes proteins containing signal peptides and other contents via fusion of secretory vesicles with the plasma membrane (PM). The conventional secretory pathway is also used by filamentous pathogens, including fungi and oomycetes to secrete effector proteins, a large proportion of which are translocated into plant cells, although the underlying mechanisms remain poorly understood. Whether plant proteins secreted through the conventional secretory pathway make their way into microbial cells is not known. Not all secreted proteins contain signal peptides; however, these proteins are secreted through unconventional secretion pathways, including as contents of extracellular vesicles (EVs).

EVs have emerged as a new route of cross-kingdom trafficking that is profoundly important in plant–pathogen interactions ^11^. A recent report convincingly demonstrated a major role for EVs in carrying sRNA cargoes for plant disease resistance ^12^. In this review, we discuss how studies on RNAi during plant–pathogen interactions have advanced our understanding of EV-mediated trafficking between plants and pathogenic microbes.

## Cross-kingdom RNA interference in plant–pathogen interactions

sRNAs, which include microRNAs (miRNAs) and small interfering RNAs (siRNAs), target complementary mRNAs or DNA (or both) to mediate post-transcriptional silencing or transcriptional silencing of target genes ^13^. RNAi was first discovered as a powerful plant defense mechanism against viruses ^14– 16^. Numerous studies in the past two decades have since shown sRNAs to be major players in plant interactions with pathogenic bacteria, fungi, and oomycetes. Plant sRNAs, Argonaute (AGO) proteins, Dicer, and Dicer-like (DCL) proteins are required for plant disease resistance to various pathogens, whereas pathogenic bacteria and oomycetes have been shown to deploy multiple effector proteins to suppress sRNA biogenesis or action in host plants ^17^. Whereas earlier studies focused on the intracellular regulatory process mediated by plant sRNAs, more recent work showed that fungal pathogen-encoded sRNAs function in silencing plant immune-related genes and enhance virulence, suggesting transportation of sRNAs from the pathogen to host cells ^6^. Conversely, plant-encoded sRNAs have also been shown to silence fungal genes to reduce pathogen virulence ^7^, supporting the notion that sRNA trafficking may be bidirectional ^9, 10^.

The gray mold fungal pathogen *Botrytis cinerea*—which infects more than 200 plant species, including *Arabidopsis* and *Solanum lycopersicum*—encodes sRNAs that are complementary to immune-related genes in the host plant ^6^. Transgenic expression of one of these sRNAs, *Bc*-siR37, silences *Arabidopsis* genes encoding a pectin lyase, a WRKY transcription factor, and a receptor-like kinase ^18^. The *B. cinerea dcl1 dcl2* double mutant strain fails to produce sRNAs and shows reduced virulence, indicating that biogenesis of sRNAs is required for pathogenesis ^6^. The *B. cinerea* sRNAs can bind to the *Arabidopsis* AGO1 protein to form an RNA-induced silencing complex (RISC), indicating that the fungus exploits host RNAi machinery to silence host genes ^6, 18^. Similarly, *Puccinia striiformis* f. sp. *tritici*, a causal agent of wheat strip rust disease, encodes an miRNA-like sRNA, termed *Pst*-milR1, that can silence a wheat gene encoding pathogenesis-related 2 ^19^.

Early studies showed that transgenic expression of artificial sRNAs complementary to root-knot nematode and insect genes in host plants can silence pest genes and enhance resistance in the plant, a phenomenon called host-induced gene silencing (HIGS) ^3, 20, 21^. Subsequent studies indicate that HIGS also provides protection against pathogenic fungi. Transgenic barley and wheat plants expressing artificial sRNAs targeted to development- and virulence-related genes of *Blumeria graminis*, a biotrophic fungal pathogen causing powdery mildew diseases in barley and wheat, show enhanced resistance to *B*. *graminis* ^5^. A similar approach has shown promise in controlling diseases caused by necrotrophic fungal pathogens. Thus, transgenic expression of sRNAs targeted to *CYP51* family genes of *Fusarium graminearum*, a necrotrophic pathogen causing deadly Fusarium head blight diseases on barley and wheat, greatly enhances disease resistance in *Arabidopsis* and barley plants ^4^. Likewise, transgenic expression of artificial sRNAs in *Arabidopsis* and tomato plants targeted to *DCL* genes of *B. cinerea* and *Verticillium dahliae*, a causal agent for wilting diseases on numerous plant species, enhances resistance to these pathogens ^22^. Furthermore, transgenic cotton plants expressing an RNAi construct targeted to *V. dahliae* hygrophobins 1 ( *VDH1*), a gene required for virulence, show enhanced resistance against *V. dahliae* infection ^23^. These studies not only provide compelling evidence for cross-kingdom trafficking of sRNAs but also provide attractive means to control diseases of great agronomic importance, as there is a paucity of Fusarium head blight resistance genes in wheat and Verticillium wilt resistance genes in cotton.

Although HIGS suggests transport of artificial sRNAs from plant cells to microbial cells, it was only recently found that endogenous sRNAs encoded by plants silence microbial genes and suppress pathogenicity. MicroRNAs miR166 and miR159 from cotton plants are induced upon infection by *V. dahliae* and their sequences are complementary to *V. dahliae* genes encoding a Ca ^2+^-dependent cysteine protease (Clp-1) and an isotrichodermin C-15 hydroxylase (Hic-15), respectively ^7^. Knockout of *Clp-1* and *Hic-15* results in reduced virulence in *V. dahliae*, indicating that Clp-1 and Hic-15 are virulence factors. Consistent with a role of the cotton miRNAs in the silencing of *Clp-1* and *Hic-15* genes in *V. dahliae*, fungal hyphae recovered from plants are significantly reduced in *Clp-1* and *Hic-15* transcripts. Importantly, miR166 is present in fungal hyphae isolated from the infected plants, providing experimental evidence that miR166 is indeed transported from plant cells to fungal cells. A more recent study showed that a number of *Arabidopsis*-derived sRNAs are delivered to *B. cinerea* cells and target to fungal genes during infection to dampen fungal virulence (see the ‘Extracellular vesicles as cargoes for cross-kingdom small RNA trafficking’ section below; ^12^). Together, these studies demonstrated that transfer of host sRNAs to fungi is an important defense mechanism for plants.

## Extracellular vesicles in host defenses and pathogenesis

EVs are broadly defined as membrane-bound vesicles released from cells. They are produced by all domains of life and generally can be classified into exosomes, shedding microvesicles, and apoptotic bodies on the basis of their size and origins ^24^. Among them, exosomes are generated by fusion of internal multivesicular bodies (MVBs) with the PM ^25^. Microvesicles are produced by directly budding from the PM ^26, 27^. Apoptotic bodies are formed only during programmed cell death ^28^. EVs function in cell–cell communication and the intercellular transport of cargoes. In animals, EVs are known to carry proteins, nucleic acids, lipids, and other compounds ^24^.

EVs are intensively studied in mammalian cells in part due to their role in modulating immune responses. Though reported in the early 1960s, plant EV studies made little progress until recent recognition of the role of EVs in plant immunity ^11, 29^. The first attempt to isolate exosome-like vesicles in plants was reported in 2009 from sunflower seeds ^30^. A subsequent study reported that EVs from sunflower seedlings are taken up by *Sclerotinia sclerotiorum* spores and cause severe growth defects in the fungus, suggesting that EVs are involved in plant immunity ^31^. More than 200 proteins were identified from these EVs, 47% of which are predicted to be cell wall–related proteins, suggesting that EVs play a role in cell wall remodeling ^32^. It is noteworthy that cell wall–related proteins were also detected in *Arabidopsis* EVs purified by the density gradient method ^33^. These results are consistent with an early electron microscopy study showing that MVBs are associated with papillae formation during powdery mildew fungal infection in barley ^34, 35^ and lend further support for a role of EVs in cell wall re-enforcement during defenses.

It has been reported that more than 50% of proteins identified in the plant secretome are leaderless and are likely secreted through unconventional secretion pathways ^36^. Unconventional protein secretion in plants is thought to be mediated by MVBs and exocyst-positive organelles ^37^, both of which are proposed to be origins of plant EVs ^38^. Rutter and Innes ^33^ also found that 84% of the proteins in the EV proteome are devoid of predicted signal peptides and this is consistent with the notion that EVs function in the unconventional protein secretion pathway.

A recent study showed that the onset of immunity is associated with increased EV production in plants ^33^. Proteins involved in biotic and abiotic stress responses are highly enriched in EVs purified from the apoplastic fluids of *Arabidopsis* plants ^33^. Among them, membrane trafficking-related protein PEN1, defense regulator RIN4, and several RIN4-interacting proteins are included ^33^. However, the proteome of EVs appeared to show little change in response to *Pseudomonas syringae* infection. It should be cautioned that some immune-related proteins may fall below the detection limit because of low abundance. In addition, it remains to be determined whether immune induction affects EV contents other than proteins.

The aforementioned studies have brought plant EVs back to researchers’ attention and plant EVs have been reviewed in detail ^11, 38, 39^. Yet another question remains to be answered. During plant–pathogen interactions, while plants secrete EVs to pathogen as a defense measure, it is also likely that pathogens pay back in kind—that pathogens also secrete EVs to plant cells to deliver their virulence factors. Indeed, Gram-negative bacteria are known to produce EVs of outer membrane origin, hence referred to as outer membrane vesicles (OMVs) ^40^. Emerging evidence shows that Gram-positive bacteria and fungi can also secrete EVs of comparable sizes with similar function as OMVs ^41^. Cargoes of pathogen EVs include virulence proteins, nucleic acids, toxins, and lipopolysaccharides ^40, 41^.

Most studies on pathogen EVs have been carried out in animal–bacterial systems. Plant pathogen EVs are assumed to function similarly to their counterparts in animal pathogens and mediate cell-to-cell communication, virulence, and modulation of plant immunity. Only a handful of studies pertaining to plant pathogen EVs have been reported. Proteomic analyses on OMVs of plant pathogenic bacteria, including *Xanthomonas campestris*, *P. syringae*, and *Xylella fastidiosa*, identified virulence-associated proteins ^42– 45^. In addition to delivering virulence-associated proteins, OMVs have been found to carry immunogenic bacterial patterns, including EF-TU and flagellin ^46^. Consistent with this, treatment with OMVs can activate defense responses in *Arabidopsis* plants ^47^. How these bacterial patterns encased inside the vesicles get detected by plant cell surface receptors remains unknown. The OMV production rate and protein composition are regulated under different growth conditions ^44^. Given that biogenesis of plant EVs also increases in response to biotic stresses, it is possible that, once confronted with each other, both plants and pathogenic bacteria concentrate their firepower by rapidly transporting cargoes to the battlefield via EVs.

The isolation and characterization of EVs from plant pathogenic fungi have not been documented to date. However, EVs are known to mediate export of fungal pathogen RNAs to human cells ^48^. It is highly likely that plant pathogenic fungi also transfer sRNAs via EVs to host cells (see the next section). Cryo-fixation transmission electron microscopy has revealed membrane-bound vesicles in the extra-haustorial matrix of *Golovinomyces orontii* in infected *Arabidopsis* leaves. It will be interesting to determine whether these vesicles are derived from fungal cells ^49^.

## Extracellular vesicles as cargoes for cross-kingdom small RNA trafficking

The phenomenon of cross-kingdom RNAi raises a question as to how sRNAs travel across the boundaries between different organisms. A recent study by Jin and colleagues convincingly showed that *Arabidopsis* cells can secrete EVs to transfer sRNAs ^12^. These secreted vesicles are taken up by *B. cinerea* cells and result in silencing of fungal genes critical for pathogenicity ^12^.

By taking advantage of different cell wall compositions, sequential protoplast purification was deployed to isolate pure fungal cells from infected tissues. sRNA profiling of purified *B. cinerea*

protoplasts identified 42 *Arabidopsis* sRNAs, 21 of which have predicted target genes in *B. cinerea*. Thirty-one of the 42 *Arabidopsis* sRNA species carried by *B. cinerea* were also found in vesicles from apoplastic fluids of infected leaves, suggesting that plant-encoded sRNAs are transferred into fungal cells via EVs. It is important to note that *Arabidopsis* sRNAs targeted to EVs are devoid of some of the most abundant sRNAs, indicating that the process is selective.

Intercellular transfer of miRNAs in animals occurs through exosomes derived from MVBs. Jin and colleagues also showed that *Arabidopsis* MVBs fuse with the PM and release EVs at the site of infection ^12^, a result confirming previous findings made in barley ^34, 35^. Isolated *Arabidopsis* EVs can be taken up by *B. cinerea* cells *in vitro* and this enables incorporation of *Arabidopsis* sRNAs into the fungal cell. In support of a role for EVs in *Arabidopsis* disease resistance to *B. cinerea*, the authors showed that loss-of-function mutations in the *Arabidopsis TET8* and *TET9* genes, which encode tetraspanin-like proteins associated with exosomes, lead to enhanced susceptibility to *B. cinerea.* These results elegantly demonstrated for the first time that, as previously speculated, EVs function as the transport vehicle for plant sRNAs and are crucial for plant immunity ^33, 39^. This study, together with a previous study made in animals ^50^, indicates that EV-mediated cross-kingdom trafficking of sRNAs is a universal defense mechanism.

Although it remains to be shown whether *B. cinerea* sRNAs are similarly transferred via EVs to plant cells, Jin and colleagues found that 7 out of 32 *B. cinerea* genes targeted by the 21 transferred *Arabidopsis* sRNAs are related to vesicle trafficking pathways. Transgenic *Arabidopsis* plants overexpressing two of these sRNAs displayed enhanced resistance, whereas knockdown of these two sRNAs led to increased susceptibility to *B. cinerea* ^12^. These exciting findings highlight the importance of vesicle trafficking in fungal virulence and open the door to future studies of sRNA trafficking from the pathogen to plant cells.

## Conclusion and perspective

Plant–pathogen interactions involve extensive exchange of molecular ammunition. Of note, cross-kingdom RNAi is an efficient strategy of attack/counterattack, as it can accurately target the enemy at a vital point. EVs serve as the armored vehicle to escort weapons to the frontline and protect cargoes from degradation by RNases and proteases in extracellular spaces.

The new advances in cross-kingdom RNAi and EVs shed light on new avenues in disease control in crop plants. A new technique, termed spray-induced gene silencing, which involves double-stranded RNAs (dsRNAs) and sRNAs that target essential pathogen genes, has shown promise in crop protection ^51– 53^. This new generation of “RNA fungicides” can remain effective for only 8 to 10 days when applied naked to plants ^22^. A recent study showed that dsRNA loaded on the layered double hydroxide (LDH) clay nanosheets (termed BioClay) extended the effectiveness to at least 20 days ^54^. Nanovesicles (NVs) mimicking EVs may be developed into an ideal vector to deliver these sRNAs ^55^. Although NVs have yet to be applied to plants, it can be envisioned that NVs loaded with specific sRNAs, defense-related proteins, or compounds targeting specific pathogens or pests may become powerful biocides.

At present, our understanding of the EV-mediated cross-kingdom traffic is just the tip of the iceberg. A number of pressing questions remain. What are the sorting mechanisms of cargo selected for export from the donor? How are the sorting mechanisms regulated in response to environmental inputs? Are defense proteins and sRNAs loaded into the same vesicles or are there EVs specific for each? How do vesicles traverse the PMs and cell walls of the plant and pathogen? The aforementioned sRNA trafficking via EVs provides an excellent model system to unlock the secret of cross-kingdom trafficking during plant–pathogen interactions.
